# Fibronectin Is a Likely Therapeutic Target Shared by Oral and Breast Carcinomas

**DOI:** 10.3390/ijms27031148

**Published:** 2026-01-23

**Authors:** Silvia Pomella, Roberto Bei, Ombretta Melaiu, Giovanni Barillari

**Affiliations:** 1 Department of Clinical Sciences and Translational Medicine, University of Rome Tor Vergata, 00133 Rome, Italy or silvia.pomella@opbg.net (S.P.); bei@med.uniroma2.it (R.B.); ombretta.melaiu@uniroma2.it (O.M.); 2 Department of Hematology and Oncology, Cell and Gene Therapy, Bambino Gesù Children’s Hospital, IRCCS, 00146 Rome, Italy; 3 School of Medicine, Saint Camillus International University of Health and Medical Sciences, 00131 Rome, Italy

**Keywords:** EMT, tumor microenvironment, macrophages, fibronectin, OSCC, breast cancer

## Abstract

The tightly controlled and transient acquisition of a motile phenotype by otherwise static epithelial cells (epithelial–mesenchymal transition, EMT) enables the repair of a damaged epithelium. Conversely, a persistent, dysregulated, and exacerbated EMT characterizes epithelial malignancies such as breast carcinoma (BC) and oral squamous cell carcinoma (OSCC), being key for their metastasis and for their escaping anti-tumor immune responses. Herein, we investigated the relationship between EMT signatures and immune cell infiltration across OSCC and metastatic BC with the aim to identify prognostic markers and/or therapeutic targets common to both these malignancies, or unique to OSCC or BC. To this end, we analyzed publicly available transcriptomic datasets to identify coding genes involved in EMT with strong correlation to immune cell signatures. The methodology consisted of data selection, correlation analysis, signature overlap determination, and validation using independent databases. Results indicated that in both OSCC and BC the expression of EMT-related genes is strongly associated with that of immunosuppressive and pro-tumor macrophages. Notably, the *FN1* gene coding for the extracellular matrix glycoprotein fibronectin (FN) emerged as the EMT gene common to either tumor types. In confirmation of this, FN protein levels were higher in OSCC and BC tissues than in their normal counterparts. Given FN capability of favoring tumor invasion and metastasis while hindering antitumor immune responses, these data encourage the development of FN antagonists to be used as an adjunct to conventional therapy in the treatment of both OSCC and BC.

## 1. Introduction

Epithelial-to-mesenchymal transition (EMT) is a multistep process by which epithelial cells lose their static and apical–basal-oriented phenotype to acquire a motile, mesenchymal-like one [1].

During the repair of a damaged epithelium, a tightly controlled EMT allows the migration of survived epithelial cells to close the wound margins; as soon as the wound is healed, however, the EMT phenotype reverts to fully epithelial [2].

While such a transient cellular plasticity governs physiologic tissue repair, a persistent, exacerbated and/or dysregulated EMT characterizes carcinomas, playing an important role in the onset and progression of these epithelial malignancies [3].

In both normal and neoplastic epithelia, EMT is triggered by inflammatory cytokines and growth or angiogenic factors that are released by tissue-infiltrating leukocytes and macrophages, or by stromal cells such as fibroblasts [4,5,6,7,8,9]. Noteworthy, in carcinomas the neoplastic cells also produce the above-mentioned cytokines, and this highly increases the concentration of EMT promoters in the microenvironment [10].

Upon their release, cytokines bind to receptors on the plasma membrane of normal or tumor epithelial cells: this is followed by the activation of intracellular signaling pathways (e.g., PI3K-AKT and/or ERK-MAPK) which, in turn, spark transcription factors such as SNAI, TWIST, and ZEB [1,2,3,6,10]. In this milieu, epigenetic modifications of ZEB, SNAI, and TWIST (e.g., DNA methylation, histones post-translational modifications, etc.) and/or microRNAs can modulate the EMT process by agonizing, or antagonizing, the transcriptional activity of ZEB, SNAI, or TWIST [11]. The latter activate the expression of genes coding for molecules that are proper of mesenchymal cells: among those gene products are vimentin (VIM, a cytoskeletal protein that confers migratory capacity to the cells that express it) [12] and proteolytic enzymes (e.g., the matrix metalloproteinases, MMPs) that degrade the extracellular matrix (ECM) and basement membranes [13]. At the same time, SNAI, TWIST, or ZEB repress the expression of epithelial molecules such as the epithelial cadherin (E-cadherin) that mediates the adhesion between neighboring epithelial cells to form lining epithelia [1,2,3,6,10]. Because of E-cadherin downregulation and the concomitant induction of VIM and ECM-degrading proteases, when undergoing EMT epithelial cells acquire migratory and invasive capabilities [1,2,3,6,10].

These phenomena are very intense and perduring in carcinomas, where they favor tumor cell invasion, migration, and, thereby, tumor metastasis [3]. In addition, EMT implies the activation of anti-apoptosis pathways: this increases cancer cell viability [14,15], not only further facilitating cancer metastasis, but also rendering tumors resistant to therapy [16,17,18,19,20,21,22]. Finally, mesenchymal-like tumor cells attract immunosuppressive cells, such as regulatory T cells, tumor-associated macrophages, and myeloid-derived suppressor cells (MDSCs) [23,24,25,26,27,28,29]. Moreover, when they undergo EMT, carcinoma cells express immune checkpoint inhibitors such as the programmed death-ligand 1 (PD-L1): the latter binds to the programmed cell death 1 receptor expressed by cytotoxic T lymphocytes, which are then functionally inhibited [30]. All this causes the tumor to escape the surveillance of the immune system and grow [23,24,28,29].

In agreement with these findings, carcinomas where cells displaying the EMT phenotype are numerous have a worse prognosis than those where EMT cells are rare or absent [3].

Based on EMT importance in carcinogenesis, here we have evaluated whether carcinomas that are different from each other in terms of onset site show an EMT signature highlighting common markers and, therefore, common targets against which to develop therapeutic tools. To this end, we have performed molecular profiling, which is an effective method for the discovery of new tumor genes and a better classification of cancer patients.

As tumor models to analyze, we have selected two highly prevalent carcinomas: breast carcinoma (BC), the most frequent malignancy in women worldwide [31], and oral squamous cell carcinoma (OSCC), which accounts for over 90% of oral cancers [32].

These two carcinoma types were chosen in view of previous studies that highlighted the similarities existing between BC and OSCC both in terms of clinical-biological behavior and carcinogenesis mechanisms [32,33,34]. Specifically, BC and OSCC are both characterized by tumor cell EMT that is associated with increased cancer invasiveness and diminished antitumor immune responses [16,17,18,19,20,21,22,35,36,37,38,39]. In this regard, one should consider that the same EMT-linked and ECM-degrading proteolytic enzymes (e.g., the MMPs, plasminogen activators, and cathepsins), as well as their interaction with membrane receptors such as CD147, modulate the invasiveness and metastatic spreading of both BC cells and OSCC cells [40,41]. Furthermore, the immunoevasion of either OSCC or BC is favored by the complement restriction factors CD46, CD55, and CD59 [42]. Moreover, BC and OSCC share molecular mediators of peritumoral inflammatory infiltration, tumor-associated lymphatic and blood vessel neoformation, and tumor growth and metastasis [33]. Additional molecular pathways involved in the development of both OSCC and BC include the zinc-alpha2-glycoprotein (a regulator of energy homeostasis and glucose/lipid metabolism), the calprotectin (a Ca^2+^ binding protein), and the serine/threonine kinase 32B (that is involved in stress and defense response) [33,43,44,45,46,47,48]. Additionally, one should consider that the development and progression of BC and OSCC are influenced by mutations in the same genes (e.g., BRCA1 and BRCA2) [49] and by steroid hormones [50]. A further shared feature between OSCC and BC is HER2/neu polymorphism [51,52].

Despite advances in diagnosis and treatment, OSCC and BC remain challenging to manage, and this highlights the need for novel therapeutic targets [16,17,18,19,20,21,22,53,54].

Surely, a greater understanding of the mechanisms that underly the interaction between EMT and the anti-OSCC and anti-BC immune response could help identify therapeutic targets common to, or exclusive to, the two tumor types. This would aid in better stratification of patients for personalized therapeutic approaches.

**Figure 1:**
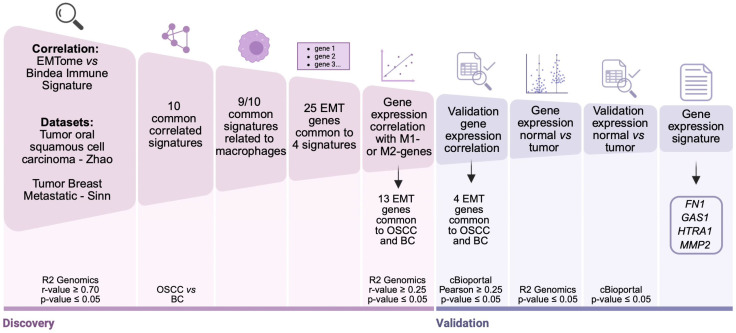
Graphical representation and summary of the cross-cancer analysis identification of EMT-associated immune gene signatures and their validation. Correlation analyses between EMTome and Bindea immune gene signatures were performed in OSCC (Zhao dataset) and BC (Sinn dataset). Ten commonly correlated immune signatures were identified, nine of which were macrophage related. From these, 25 EMT genes were shared across four correlated signatures. Expression levels of these genes were further analyzed for correlation with M1- and M2-like macrophage markers. Validation was conducted using R2 Genomics and cBioPortal platforms, applying statistical thresholds of *r*-value > 0.25 and *p*-value < 0.05. Comparative expression analysis between normal and tumor samples revealed 13 EMT genes shared between OSCC and BC, with FN1, GAS1, HTRA1, and MMP2, consistently differentially expressed. Created in https://BioRender.com (accessed on 23 September 2025).

In the present study, we have employed a multitiered computational framework to investigate the relationship between EMT signatures and immune cell infiltration across OSCC and metastatic BC. Publicly available transcriptomic datasets have been analyzed to identify EMT-related coding genes with strong correlation to immune cell signatures. The methodology consists of data selection, correlation analysis, signature overlap determination, and validation using independent databases (Figure 1).

## 2. Results

### 2.1. Correlation Between EMT- and Macrophage-Signature in OSCC and BC Patients

**Figure 2:**
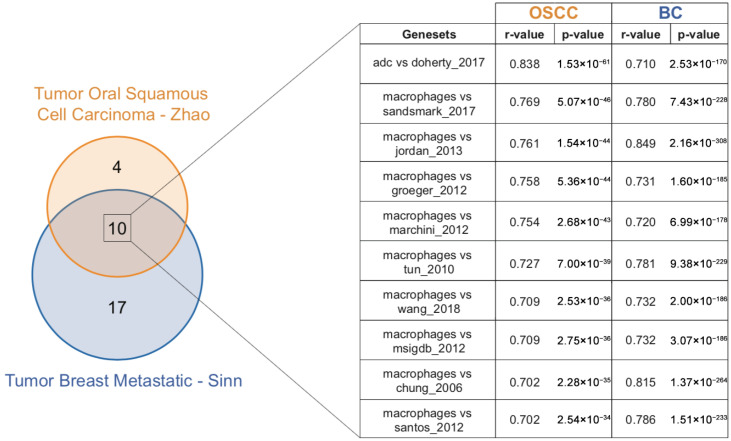
Expression correlation between EMTome signature and Bindea Immune Signature Panel in OSCC and BC patients. Gene expression correlations between the EMTome signature and the Bindea Immune—Signature Panel were computed using two tumor datasets: OSCC (Zhao; *n* = 229; MAS5.0; u133p2) and metastatic BC (Sinn; *n* = 1108; MAS5.0; u133a). Correlations were considered significant with a *r*-value ≥ 0.7 and *p*-value ≤ 0.05, and the resulting associations between EMT and immune-related gene signatures were reported.

To investigate the relationship between EMT and immune modulation in patients with OSCC (Tumor Oral Squamous Cell Carcinoma–Zhao–229–MAS5.0–u133p2) or BC (Tumor Breast Metastatic–Sinn–1108–MAS5.0–u133a), we computed expression correlations between the EMTome signature [55], a publicly available repository of 84 EMT-related signatures, and the Bindea Immune Signature Panel [56], which includes immune cell-type signatures from 24 immune cell subsets (i.e., M1-like/M2-like macrophages, CD8^+^ T cells, natural killer [NK] cells). Applying thresholds of correlation coefficient *r*-value ≥ 0.7 and *p*-value ≤ 0.05, we identified 14 significant positive EMT–immune signature correlations in the OSCC dataset and 27 in the BC dataset (Figure 2 and Supplementary Table S1). Notably, 11 out of the 14 correlated immune signatures in OSCC and 23 out of 27 in BC involved macrophage-related immune populations. To compare EMT-associated immune modulation between OSCC and BC, we identified 10 overlapping EMT–immune signature pairs, 9 of which were linked to macrophage signatures, suggesting a strong and consistent association between EMT activation and macrophage polarization. To further explore the genes potentially driving this association, we retrieved and compared gene lists from the 10 shared EMT signatures. Among the 447 unique genes identified, 25 were shared by at least 4 of the EMT signatures (*CDH11*, *CDH2*, *COL3A1*, *COL5A1*, *COL6A1*, *COL6A2*, *COL6A3*, *CTGF*, *CXCL1*, *DCN*, *FN1*, *GAS1*, *HTRA1*, *INHBA*, *MMP2*, *PCOLCE*, *PDGFRA*, *PDGFRB*, *PMP22*, *SPARC*, *STAT1*, *TGM2*, *TNC*, *VIM*, and *WNT5A*), highlighting a set of recurrent EMT-associated genes potentially involved in immune modulation.

### 2.2. EMT Genes Positively Correlate with M2-like Macrophage Genes in Patients with OSCC and BC

To investigate the macrophage subtypes associated with EMT-related transcriptional programs, we analyzed the expression correlation between twenty-five EMT-associated genes and key macrophage markers. Specifically, we examined *CD163*, a scavenger receptor commonly used as a marker of alternatively activated M2-like tumor-associated macrophages known for their immunosuppressive and pro-tumoral functions [57]. We also included the triggering receptor expressed on myeloid cells 2 (*TREM2*), a receptor expressed by lipid-associated macrophages, playing a critical role in immune evasion, tumor progression, and metabolic adaptation within the tumor microenvironment [58,59,60]. To represent the classically activated M1-like anti-tumoral macrophage phenotype, we assessed inducible nitric oxide synthase (*NOS2*), an enzyme mediating pro-inflammatory and cytotoxic responses [61], and *CD68*, a macrophage lysosomal glycoprotein [62].

**Figure 3:**
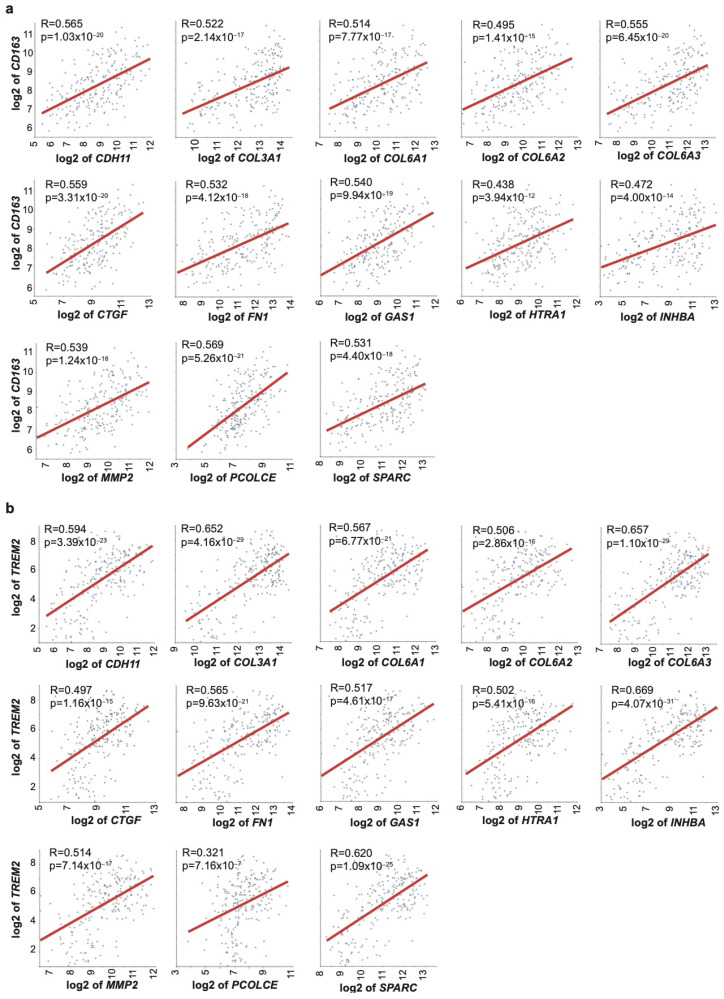
Gene expression correlation of 13 EMT genes with M2-like macrophages markers in OSCC patients. Gene expression correlation has been computed in OSCC dataset (Zhao; *n* = 229; MAS5.0; u133p2) between 13 EMT genes and (**a**) *CD163* and (**b**) *TREM2*. Red lines represent best-fit linear regressions. Exact *r*-values and *p*-values are reported in the figure.

Applying thresholds of correlation coefficient *r* ≥ 0.25 and *p* ≤ 0.05, we identified twenty-four EMT-related genes that exhibited statistically significant positive correlations with *CD163* in OSCC (Tumor Oral squamous cell carcinoma–Zhao–229–MAS5.0–u133p2), suggesting a strong transcriptional association between mesenchymal programming and the presence of immunosuppressive macrophages (Figure 3a and Supplementary Table S2). The strongest correlations were observed for procollagen C-proteinase enhancer protein (*PCOLCE*, *r* = 0.569), connective tissue growth factor (*CTGF*, *r* = 0.559), the alpha 3 chain of collagen type VI (*COL6A3*, *r* = 0.555), fibronectin 1 (*FN1*, *r* = 0.532), secreted protein acidic and rich in cysteine (SPARC, *r* = 0.531), MMP2 (*r* = 0.539), Growth Arrest Specific 1 (*GAS1*, *r* = 0.540), the alpha 1 chain of collagen type III (*COL3A1*, *r* = 0.522), inhibin beta A subunit (*INHBA*, *r* = 0.472), and high-temperature requirement A1 (*HTRA1*, *r* = 0.438).

A complementary analysis revealed similarly strong positive correlations between 19 EMT genes and *TREM2*, reinforcing the connection between EMT and a macrophage-enriched tumor microenvironment. Top correlated genes included *INHBA* (*r* = 0.669), *COL6A3* (*r* = 0.657), *COL3A1* (*r* = 0.652), *SPARC* (*r* = 0.620), cadherin 11 (*CDH11*, *r* = 0.594), the alpha 1 chain of collagen VI, (*COL6A1*, *r* = 0.567), *FN1* (*r* = 0.565), *GAS1* (*r* = 0.517), the alpha 2 chain of collagen type VI (*COL6A2*, *r* = 0.506), *HTRA1* (*r* = 0.502), *CTGF* (*r* = 0.497), and *PCOLCE* (*r* = 0.321) (Figure 3b and Supplementary Table S2).

In contrast, NOS2 showed no significant correlations with EMT genes in OSCC, consistent with a lack of association between EMT and anti-tumoral macrophages. Furthermore, *CD68* expression data were not available for OSCC, limiting the assessment of general macrophage presence in this context (Supplementary Table S2).

**Figure 4:**
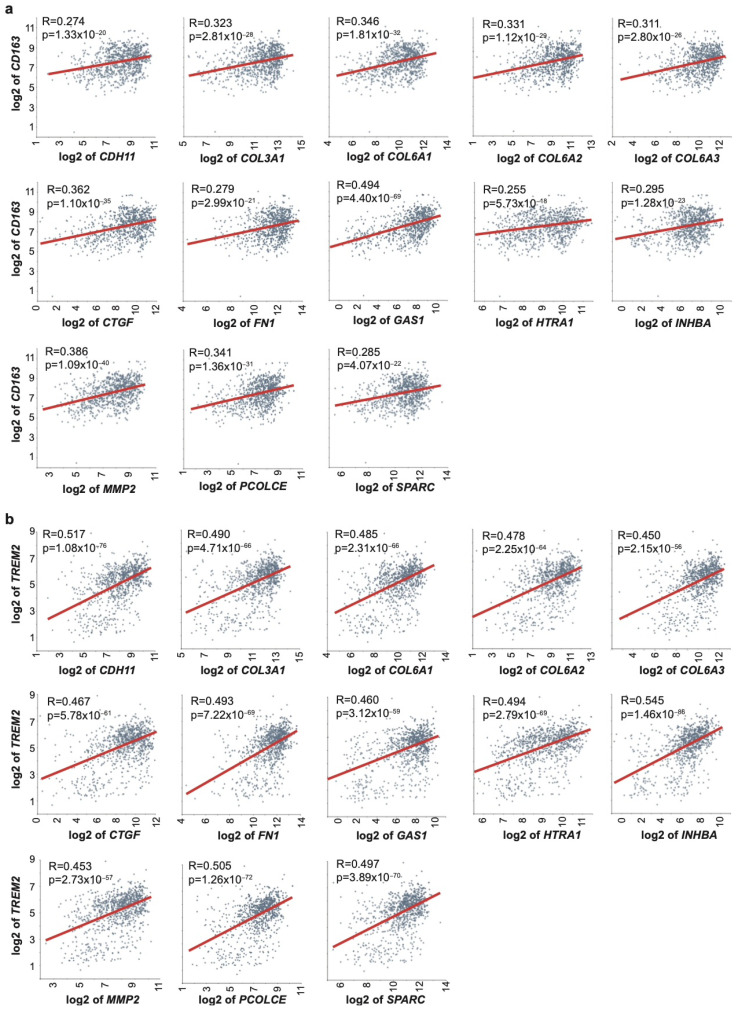
Gene expression correlation of 13 EMT genes with M2-like macrophages markers in BC patients. Gene expression correlation has been computed in BC dataset (Sinn; *n* = 1108; MAS5.0; u133a) between 13 EMT genes and (**a**) *CD163* and (**b**) *TREM2*. Red lines represent best-fit linear regressions. Exact *r*-values and *p*-values are reported in the figure.

A similar pattern was observed in the BC dataset (Tumor Breast Metastatic–Sinn–1108–MAS5.0–u133a). For *CD163*, 18 EMT genes showed positive correlations (*r* = 0.255–0.494), with the highest associations for *GAS1* (*r* = 0.494), *CTGF* (*r* = 0.362), and *PCOLCE* (*r* = 0.341) (Figure 4a and Supplementary Table S2). Correlations with *TREM2* were stronger, with 20 EMT genes meeting the significance threshold (*r* = 0.450–0.545); top genes included *INHBA* (*r* = 0.545), *PCOLCE* (*r* = 0.505), *SPARC* (*r* = 0.497), and *CTGF* (*r* = 0.467) (Figure 4b and Supplementary Table S2). As in OSCC, *NOS2* did not correlate significantly with EMT genes, and only the genes coding for *MMP2*, platelet-derived growth receptor alpha (*PDGFRA*), transglutaminase 2 (*TGM2*), or *VIM* showed weak correlations with *CD68* (*r* = 0.251–0.306; Supplementary Table S3).

Finally, we identified a core set of 13 EMT genes—*CDH11*, *COL3A1*, *COL6A1*, *COL6A2*, *COL6A3*, *CTGF*, *FN1*, *GAS1*, *HTRA1*, *INHBA*, *MMP2*, *PCOLCE*, and *SPARC*—consistently correlated with both *CD163* and *TREM2* in both the OSCC and BC datasets. To validate our initial findings, we assessed gene expression correlations in independent cohorts from TCGA (PanCancer Atlas) for Head and Neck Squamous Cell Carcinoma and Breast Invasive Carcinoma. Among the 13 EMT-related genes evaluated, 11 genes—*CDH11*, *COL3A1*, *COL6A1*, *COL6A2*, *COL6A3*, *FN1*, *GAS1*, *HTRA1*, *MMP2*, *PCOLCE*, and *SPARC*—demonstrated significant positive correlations, thereby corroborating the associations observed in the discovery datasets (Supplementary Figure S1 and Table S4). These findings collectively support a strong association between EMT-related transcriptional programs and the presence of immunosuppressive macrophages within the tumor microenvironment. The consistent lack of correlation with M1-like markers such as *NOS2*, along with limited association with *CD68*, suggests that EMT activation preferentially aligns with macrophage phenotypes that promote immune evasion and tumor progression.

### 2.3. Shared and Tumor-Specific EMT-Immunogenic Gene Signatures in OSCC and BC

**Figure 5:**
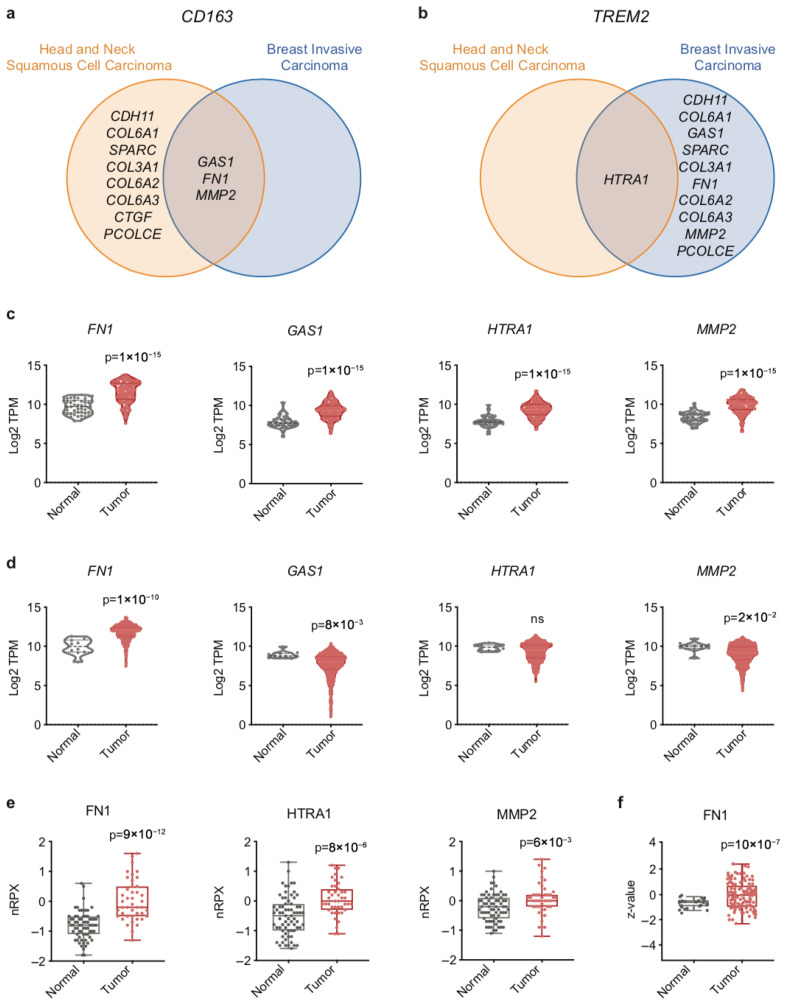
Expression of EMT genes and proteins in normal and tumor tissues of OSCC and BC patients. Venn diagram-based analysis and gene list intersection for (**a**) *CD163* and (**b**) *TREM2* were used to determine shared EMT-immunogenic relationships between OSCC and BC datasets. (**c**) Gene expression levels of *FN1*, *GAS1*, *HTRA1*, and *MMP2* were compared between normal (n = 45) and OSCC tissues (*n* = 167) (Zhao cohort). (**d**) Gene expression levels of *FN1*, *GAS1*, *HTRA1*, and *MMP2* were compared between normal (*n* = 10) and BC tissues (*n* = 868) (Sinn cohort). (**e**) Protein expression levels of FN, HTRA1, and MMP2 were compared between normal (*n* = 70) and OSCC tissues (*n* = 42) (CPTAC cohort). (**f**) Protein expression levels of FN were compared between normal (*n* = 18) and BC tissues (*n* = 125) (CPTAC cohort). Exact *p*-values, calculated using two-tailed unpaired *t*-test, are reported in the figure. TPM: transcript per million; nRPX: normalized relative protein expression.

To identify shared EMT-immunogenic relationships between OSCC and BC, we performed gene list intersection analyses focusing on *CD163* and *TREM2*-associated EMT genes. Using Venn diagram-based comparisons, we found that three EMT-related genes—*FN1*, *GAS1*, and *MMP2*—were positively correlated with *CD163* in both OSCC and BC datasets (Figure 5a). Additionally, *HTRA1* was the only EMT gene positively correlated with *TREM2* across both tumor types (Figure 5b).

To further evaluate the relevance of these four EMT genes (*FN1*, *GAS1*, *HTRA1*, and *MMP2*), we examined their transcript expression in tumor versus normal tissue. In the Zhao OSCC cohort, all four genes showed significant upregulation in tumor samples compared to normal tissue (95% confidence intervals (CI) *FN1* [1.5, 2.3]; *GAS1* [1.1, 1.8]; *HTRA1* [1.2, 1.8] and *MMP2* [1.3, 1.9]) (Figure 5c). These findings were validated using the OSCC cohort from the TCGA PanCancer Atlas cohort for Head and Neck Squamous Cell Carcinoma, where all four genes again exhibited elevated expression in tumors relative to normal samples (Supplementary Figure S2a). Furthermore, stratification of OSCC samples by histological grade showed that *FN1* expression increased predominantly in high-grade tumors, whereas *GAS1* and *MMP2* were significantly elevated across multiple grades when compared to normal tissue (Supplementary Figure S3a). Conversely, *HTRA1* expression displayed a non-monotonic pattern, with levels in grade 4 tumors returning to values comparable to normal tissue (Supplementary Figure S3a). Analysis by tumor stage revealed significant differences in expression for all four genes across stages, without a consistent linear trend (Supplementary Figure S3c).

Analysis of the BC dataset (Sinn cohort) revealed differential regulation: *FN1* was significantly upregulated in tumor samples (95% CI *FN1* [1.3, 2.5]), whereas *GAS1* and *MMP2* were downregulated, and *HTRA1* did not show a significant difference (Figure 5d). These patterns were corroborated in the TCGA PanCancer Atlas Breast Invasive Carcinoma cohort (Supplementary Figure S2a). Stratification of BC samples by intrinsic molecular subtypes showed that *FN1* expression differed significantly across tumor types, with higher levels observed in HER2-enriched, Luminal A, and Luminal B tumors compared to other subtypes (Supplementary Figure S3b). Similarly, analysis based on tumor stage revealed significant differences in *FN1* expression across several stages compared to normal tissue, without evidence of a consistent stage-dependent trend (Supplementary Figure S3d). Protein expression analysis using CPTAC data showed consistent trends. In OSCC samples, FN, MMP2, and HTRA1 proteins were all significantly upregulated in OSCC compared to normal tissues (Figure 5e). No protein data were available for GAS1. In BC, only FN protein levels were elevated in tumors (Figure 5f).

Our findings reveal both shared and tumor-specific EMT-immunogenic gene associations in OSCC and BC, highlighting a conserved four-gene EMT signature (*FN1*, *GAS1*, *HTRA1*, and *MMP2*) in OSCC, and identifying *FN1* as the EMT gene common to both tumor types.

## 3. Discussion

Herein, we have investigated the relationship between EMT and the features of the immune microenvironment of OSCC and BC, two highly prevalent carcinomas that differ under various aspects, primarily for the site of development, but share common molecular mechanisms leading to their onset and progression.

Our results have indicated that the *CD163* scavenger receptor and *TREM2*, both markers of pro-tumor macrophages [57,59,60], are expressed in OSCC and BC, and that connections exist in OSCC or BC between the expression of these M2-like macrophage markers and that of coding genes involved in EMT [57,59,60]. Specifically, results from gene correlation analyses have identified 13 EMT-related genes that are significantly associated with the expression of both *CD163* and in *TREM2* in OSCC and BC. Conversely, we have detected in OSCC or BC no significant association between the expression of EMT-related genes and that of *NOS2*, a marker of classically activated, proinflammatory, and cytotoxic M1-like macrophages [61].

Confirmatory work performed by examining TCGA cohorts of Head and Neck Squamous Cell Carcinoma and Breast Invasive Carcinoma has revealed that out of the thirteen previously identified EMT genes, eleven—*CDH11*, *COL3A1*, *COL6A1*, *COL6A2*, *COL6A3*, *FN1*, *GAS1*, *HTRA1*, *MMP2*, *PCOLCE*, and *SPARC*—significantly associated with the M2-like signature in both OSCC and BC.

Ten of these eleven genes accompany the EMT process and/or are a marker of it [63,64,65,66,67,68,69,70,71]. The only exception is the gene coding for HTRA1, a serine protease countering EMT in cancer cells [72,73]. Unfortunately, we found no data on HTRA1 expression in OSCC compared to healthy oral tissue. To date, information on the role this gene may have in oral carcinogenesis appears to be limited to in vitro studies conducted on OSCC cell lines [74]. Instead, HTRA1 levels are known to be reduced or lost in invasive BCs as compared to normal breast tissue, and to be much lower in BC tumor cells than in the non-tumor ones [75]. Coherently, HTRA1 has been shown to inhibit the EMT, migration, and invasion of BC tumor cells [75]. In our opinion, given HTRA1 and TREM2’s capability of modulating the EMT process and the polarization of tumor-associated macrophages, respectively, the two events are intertwined over time during BC and OSCC carcinogenesis.

Some of the EMT genes that in both BC and OSCC positively correlate with the expression of tumor associated-macrophages markers code for ECM components or ECM-remodeling enzymes with a role in BC or OSCC tumorigenesis.

Specifically, COL3A1 is overexpressed in both OSCC and BC, where it promotes proliferation, invasion, migration, and immune escape of the tumor cells [76,77]. Likewise, COL6A1 is overexpressed in BCs where, together with COL6A2 and COL6A3, it contributes to the metastasis of BC cells [78]. Noteworthy, COL6A1 levels are high in the saliva of individuals with early-stage OSCC [79].

SPARC is a glycoprotein habitually present in the ECM of a tissue undergoing repair, where it remains only for a limited time to provide pro-adhesive support for migrating and proliferating cells [80]. SPARC is instead present stably and at high levels in OSCC and BC tissues, where it facilitates tumor cell invasion and metastasis [81,82,83].

*FN1* is another EMT-related ECM gene whose expression has resulted as positively correlated with that of tumor-associated macrophage genes in both OSCC and BC. *FN1* encodes FN, a glycoprotein that has a ubiquitous distribution in the human body, as it can be found on the cellular membrane, in body fluids and in the ECM, where it is deposited as fibrils and binds fibrin, tenascin, proteoglycans, and/or collagens [84]. Of note, FN is overexpressed in the stroma of a variety of carcinomas, OSCC and BC included, with little or no expression in adjacent non-tumoral areas [85,86,87,88,89]. In healthy, normal tissues, FN is synthesized by hepatocytes, epithelial cells, and fibroblasts [84]; in a physiological setting, it can be part of the provisional ECM that provides mechanical support for tissue repair [90]. In tumor tissues, FN is overproduced by cancer-associated fibroblasts or by the carcinoma cells themselves, especially when they display the EMT phenotype [88,91,92].

Regarding the ECM-degrading MMP-2, it is highly expressed in both BC and OSCC, where it promotes the invasion of tumor cells [93,94]. Conversely, the levels of PCOLCE, an enhancer of procollagens catalysis, are lower in BCs than in their normal counterparts [95], suggesting that PCOLCE may contribute to making the peritumoral ECM more permissive to cancer cell invasion. We found no information on PCOLCE and OSCC.

Other EMT-related genes that we have determined to positively correlate with *CD163* and *TREM2* expression in both BC and OSCC are those coding for the mitogenic GAS1 [96,97] or for CDH11, a receptor mediating the adhesion among mesenchymal cells [98,99]. Concerning GAS1, it triggers OSCC cell proliferation [96,97] and maintains cancer stem cells in BCs [100]. Regarding CDH11, it is overexpressed in OSCC compared to normal oral tissue [98], as well as in BC, where it facilitates tumor cell metastasis to bone [99].

The induction of tumor cell EMT is certainly attributable to the paracrine action of cytokines released by pro-tumoral macrophages. Specifically, in BC, these macrophages produce the chemokine C-C motif ligand 2 which, in turn, promotes EMT of carcinoma cells [8]. In OSCC, macrophages release interleukin (IL)-1β, IL-6, IL-8, tumor necrosis factor, and transforming growth factor (TGF)-β1, all of which induce EMT in OSCC cells, markedly enhancing their migratory and invasive potential [101].

Our work has been complemented and concluded by transcriptomic and proteomic analyses, which have identified a four-gene/protein EMT signature (FN, GAS1, HTRA1, and MMP2) in OSCC and a single-gene/protein EMT signature (FN) in BC. Specifically, OSCCs display FN, GAS1, HTRA1, and MMP2 mRNA and protein levels higher than those present in normal oral tissue. Unlike OSCCs, BCs show a decrease in GAS1 and MMP2 mRNA compared to their normal counterparts, while *HTRA1* gene expression does not change in BC compared to normal breast tissue. In BCs, only the gene and protein expression of FN are increased as compared to control tissues.

The fact that the expression of HTRA1, GAS1, and MMP2 in the BCs follows a trend opposite to that of the OSCCs requires meditation.

As for HTRA1, it must be highlighted that its expression is upregulated in a few tumor types and downregulated in others [102]. Moreover, it has to be underscored that HTRA1 levels do not significantly correlate with the stage of progression and/or degree of differentiation of all tumor types in which it has been examined [102].

Regarding GAS1, one should consider that its levels are increased in OSCCs as compared to healthy oral tissues [96,97]. Conversely, GAS1 is downregulated in advanced BCs [103]. Noteworthily, GAS1 levels in BC tissues positively correlate with cancer cell invasiveness and negatively correlate with cancer cell growth, suggesting that a reduction in GAS1 expression could promote the transition of BC cells from stationary to invasive [103]. Indeed, a soluble form of GAS1 has been found to inhibit BC growth [104,105].

Finally, it should be remembered that while it is well established that MMP-2 levels are much higher in OSCC than in healthy oral tissue [106], there are BC displaying a frankly epithelial phenotype, which express low MMP-2 levels: these BC types have a better prognosis than BC with an EMT phenotype, which overexpress MMP-2 [107,108,109].

In conclusion, among EMT-related molecules that we have found to be consistently correlated with the infiltration of either OSCC or BC by tumor-associated macrophages, FN is the only one whose mRNA and protein levels are upregulated in both OSCC and BC datasets.

In this context, one should consider that the ECM molecule FN participates in a variety of cellular processes including adhesion, migration, proliferation, and differentiation [110,111]. Such phenomena are mediated by the binding of extracellular FN to cell surface receptors belonging to the integrin family [112]. Among those receptors is alpha 5 beta 1, which recognizes the arginine glycine aspartic acid (RGD) sequence of FN and is considered as the main FN receptor [112].

The binding of FN to alpha 5 beta 1 expressed on the surface of tumor cells, OSCC and BC cells included, favors their growth, invasion, and migration [113,114,115]. In addition, the interaction between FN and alpha 5 beta 1 drives the formation of new blood vessels that nourish the growing tumor and provide it with additional metastatic routes [72]. Moreover, FN binding to the alpha 5 beta 1 expressed on the surface of macrophages triggers the polarization of the latter toward a protumor phenotype, hence creating an immunosuppressive microenvironment [116,117,118]. Finally, the large amounts of FN deposited in the peritumoral stroma combine with collagens and other ECM molecules: this creates a mechanical barrier that prevents cytotoxic T lymphocytes from reaching the tumor parenchyma [84].

Based on these findings, antagonists of the FN-alpha 5 beta 1 interaction have been developed and exploited for antitumor therapy: amidst them is cilengitide, a cyclic RGD peptide hampering the function of alpha 5 beta 1, as well as that of other RGD-binding integrins [116,119]. Noteworthy, cilengitide can block the pro-tumor polarization of BC-infiltrating macrophages promoted by FN [116]. This finding, together with the fact that both OSCC and BC cells express alpha 5 beta 1 [120], would suggest employing cilengitide in the treatment of OSCC and BC. In this context, preclinical work has demonstrated the efficacy of cilengitide against the adhesion, survival, growth, invasion, and/or therapeutic resistance of BC and OSCC cells, bolstering the addition of this drug to conventional antitumor chemo/radiotherapy [121,122,123]. This approach is further supported by the fact that the pharmacokinetics of cilengitide are well characterized, allowing its rapid translational application in OSCC or BC patients [124].

However, results from clinical trials have demonstrated that, although well tolerated, cilengitide had modest therapeutic outcomes in patients with glioblastoma multiforme [125,126] and no detectable clinical activity in patients affected by prostate carcinoma [127], head and neck squamous cell carcinoma [128], unresectable pancreatic carcinoma [129], metastatic melanoma [130], or advanced non-small-cell lung cancer [131].

Reasons underlying the lack of efficacy of cilengitide in these types of malignancies are likely to be multiple. First of all, it must be highlighted that the studies cited here have mostly recruited patients affected by aggressive tumors and in a state of clinical progression so advanced as to make them poorly responsive to therapy [127,128,129,130,131]. Furthermore, one should consider that the short half-life of cilengitide causes the plasma concentration of the drug to fall below the biologically active level just a few hours after its administration [130]. This situation is aggravated when patients, for reasons of drug availability, took cilengitide with long time intervals between one administration and the next [130]. Another likely cause for the clinical failure of cilengitide is that some of the treated tumors expressed low levels of the drug’s target integrins [128,130]. In this regard, one should also take into account that integrins other than alpha 5 beta 1 or alpha v beta 3 could confer resistance to cilengitide treatment, as suggested by previous in vitro work [132]. For example, the Arg-Glu-Asp-Val or the Leu-Asp-Val sequence of FN can bind to the alpha 4 beta 1, alpha 4 beta 7, or alpha 9 beta 1 integrins [133].

Analogously to what has occurred with cilengitide, an anti-alpha 5 beta 1 antibody has shown poor activity against ovarian cancer [84]. In addition to these disappointing clinical data, it must be highlighted that clinical studies aimed at evaluating the efficacy of antitumor chemotherapeutics conjugated with FN ligands other than antibodies have not yet provided definitive results [84]. Notwithstanding, counteracting FN-induced cellular events could still represent a valid therapeutic strategy for both OSCC and BC to be combined with more conventional treatment regimens such as chemotherapy, radiotherapy, and/or immunotherapy. Indeed, adhesion to FN reduces the sensitivity of tumor cells to anticancer chemo/radiotherapy [84]. Moreover, FN exacerbates the EMT of tumor cells, making them more aggressive [88,91,92,113,117,118,134].

In our opinion, inhibition of FN production could be a valid antitumor therapeutic approach, alternative to antagonizing or mimicking FN activity.

In this regard, it is known that the expression of FN is triggered by the activation (phosphorylation) of the signaling protein AKT [135,136]. The finding that activated AKT promotes many of the events underlying carcinogenesis has long prompted the design and development of drugs capable of inhibiting AKT activation [137,138,139]. Some of these drugs have been approved by the FDA and used in cancer therapy but have been found to have significant toxicity [137,138,139]. Actually, the development of AKT inhibitors with reduced toxicity is an important therapeutic goal that has been sought for a long time.

In this context, our recent results indicate that therapeutic doses of Ritonavir (RTV), an inhibitor of human immunodeficiency virus replication endowed with unpredicted antitumor effects, counter AKT phosphorylation in OSCC tumor cells [140]. Because of this activity, RTV reverts the EMT phenotype of OSCC cells into a more epithelial one and blocks the invasion of OSCC cells while sensitizing them to ionizing radiation [140].

Other research groups have shown that RTV inhibits the activation (phosphorylation) of AKT also in BC cells, reducing BC cell viability and increasing the activity of antitumor drugs in an animal model of BC [141,142].

RTV has been in use for many years, and its pharmacokinetics in humans are well known [143]. This, and the fact that RTV is generally well tolerated by most patients who take it, would allow the reuse of RTV in the therapy of OSCC and BC, particularly in the aggressive forms of these two types of carcinomas, which are populated by a high number of tumor cells with EMT phenotype and characterized by a stroma containing an elevated amount of FN.

A key limitation of this study is that it is based solely on publicly available datasets and lacks validation using clinical samples. Additionally, some cohorts, particularly the BC dataset, show an imbalance between tumor and normal samples. While our analytical pipeline and cross-dataset validations help strengthen the findings, these results should be interpreted with caution. Future studies including larger, balanced cohorts and clinical validation are warranted to confirm and extend these observations.

## 4. Materials and Methods

### 4.1. Signature Correlation Analysis

The correlation between EMT and immune infiltration in the OSCC dataset (Tumor Oral squamous cell carcinoma–Zhao–229–MAS5.0–u133p2) and BC dataset (Tumor Breast Metastatic–Sinn–1108–MAS5.0–u133a) has been assessed on R2 Genomics Analysis and Visualization Platform (http://r2.amc.nl, accessed on 3 June 2025). Signature expression correlations in the OSCC and BC dataset have been computed between the EMTome signature [55] and the Bindea Immune Signature Panel [56]. OSCC and BC correlations have been ranked based on *r*-value, and only results with *r*-value ≥ 0.7 and *p*-value < 0.05 have been considered.

### 4.2. Gene Correlation Analysis

The correlation between selected EMT genes and specific M1- or M2-like Macrophage genes on OSCC dataset (Tumor Oral squamous cell carcinoma–Zhao–229–MAS5.0–u133p2) and BC dataset (Tumor Breast Metastatic–Sinn–1108–MAS5.0–u133a) has been computed on R2 Genomics Analysis and Visualization Platform (accessed on 10 June 2025). Available correlations have been downloaded, and thresholds have been set for *r*-value ≥ 0.25 and *p*-value ≤ 0.05.

Validation of gene correlations on Head and Neck Squamous Cell Carcinoma and Breast Invasive Carcinoma cohorts, both from The Cancer Genome Atlas (TCGA, PanCancer Atlas), has been performed on cBioPortal for cancer genomics (https://www.cbioportal.org/, accessed on 12 June 2025). Thresholds have been applied for Pearson ≥ 0.25 and *p*-value ≤ 0.05.

### 4.3. Gene Expression Analysis

Transcriptomic data of OSCC samples (Tumor Oral squamous cell carcinoma–Zhao–229–MAS5.0–u133p2) and BC samples (Tumor Breast Metastatic–Sinn–1108–MAS5.0–u133a) have been retrieved from R2 Genomics Analysis and Visualization Platform (accessed on 20 June 2025). Data from primary normal and tumor tissues have been compared and plotted on GraphPad Prism 8.2.1 (GraphPad Software, Boston, MA, USA). Validation of gene expression comparison on Head and Neck Squamous Cell Carcinoma (TCGA, PanCancer Atlas) and Breast Invasive Carcinoma (TCGA, PanCancer Atlas) cohorts has been performed by downloading patients’ transcriptomic data from the Xena database [144]. Clinicopathological features for OSCC and BC patients have been downloaded from cBioportal for cancer genomics (https://www.cbioportal.org/, accessed on 15 June 2025). Only samples containing both RNA count data and information on the anatomic site of the neoplasm were included. OSCCs developed on the base of the tongue, on the tongue, on the oropharynx, or on the floor of the mouth were selected as in [145].

### 4.4. Protein Expression Analysis

Proteomic data of OSCC and relative normal samples have been downloaded from the HNSC CPTAC dataset of Protein Atlas portal (https://www.proteinatlas.org, accessed on 4 July 2025). Protein expression values were analyzed as provided by the CPTAC standardized processing pipeline and are reported in normalized form by the source repository. Information on the anatomic site of the neoplasm has been retrieved from the Proteomic Data Commons (https://pdc.cancer.gov/pdc/browse, accessed on 4 July 2025) and matched with protein expression values. OSCC cases were selected based on the following anatomic site of the neoplasm: base of the tongue, tongue, oropharynx, and floor of the mouth. Data have been plotted on GraphPad Prism 8.2.1. Proteomic data for BC and relative normal samples have been downloaded from the CTPAC database of UALCAN portal (https://ualcan.path.uab.edu, accessed on 6 July 2025) where protein expression levels are provided as normalized values according to the CPTAC workflow.

### 4.5. Statistical Analysis

*r*-values and *p*-values for signature correlation analysis have been retrieved from R2 Genomics Analysis and Visualization Platform. Only correlations with *r*-value ≥ 0.7 and *p*-value ≤ 0.05 have been considered. *r*-values and *p*-values for gene correlation analysis have been retrieved from R2 Genomics Analysis and Visualization Platform. Only correlations with *r*-value ≥ 0.25 and *p*-value ≤ 0.05 have been considered. Pearson and *p*-values for validation of gene correlation analysis have been retrieved from the cBioPortal for cancer genomics. Thresholds have been applied for Pearson ≥ 0.25 and *p*-value ≤ 0.05. *p*-values for gene and protein expression analysis have been calculated with GraphPad Prism 8.2.1 using two-tailed unpaired *t*-test. *p*-values ≤ 0.05 have been considered statistically significant. Statistical comparisons between tumor and normal samples for the four prioritized EMT genes (*FN1*, *GAS1*, *HTRA1*, and *MMP2*) were performed using unpaired two-tailed Student’s *t*-tests. For these comparisons, 95% confidence intervals (CIs) for the mean differences were also calculated to provide complementary information on effect size and variability. Exact *r*-values, Pearson, and *p*-values are reported in the figures.

## 5. Conclusions

This study presents a comparative cross-cancer analysis of EMT-associated immune interactions in OSCC and BC, revealing a robust association between EMT transcriptional programs and immunosuppressive macrophages in both malignancies. By integrating multiple publicly available transcriptomic and proteomic datasets, FN1 has emerged as a shared core EMT-related gene with high expression in tumor tissues, being associated with malignant tumor phenotypes. These findings provide a rationale for targeting FN as a common therapeutic strategy in OSCC and BC.

Notwithstanding the stringency of the analytical pipeline, this study has inherent limitations. The analyses are based on in silico data and lack direct validation in clinical specimens, and although the multi-layered approach partially mitigates this limitation, confirmation in larger and independent patient cohorts is warranted. Nevertheless, our findings are supported by previous studies on OSCC and BC clinical samples demonstrating a positive correlation between FN expression levels and tumor aggressiveness.

These clinical observations, consistent with the known role of FN in promoting tumor growth and metastatic dissemination, have previously driven the development of therapeutic agents targeting FN–integrin interactions through the RGD motif. However, such approaches have shown limited clinical efficacy, likely due to the ability of FN to exert biological effects through non-RGD domains and integrin-independent mechanisms.

In this context, we propose that therapeutic strategies aimed at reducing FN synthesis may represent a more effective approach to limiting OSCC and BC progression. In particular, inhibition of the AKT signaling pathway appears promising, as AKT activation not only promotes FN production but also enhances tumor cell proliferation, invasion, survival, and resistance to both cytotoxic chemotherapy and ionizing radiation. Taken together, these findings provide a strong rationale for integrating conventional chemotherapy and radiotherapy with AKT-targeting agents as part of combinatorial therapeutic strategies designed to restrain tumor progression and restore antitumor immune responses in OSCC and BC.

## Data Availability

The original contributions presented in this study are included in the article/Supplementary Material. Further inquiries can be directed to the corresponding author.

## Supplementary Materials

The following supporting information can be downloaded at https://www.mdpi.com/article/10.3390/ijms27031148/s1.

## Abbreviations

The following abbreviations are used in this manuscript:

BC	Breast carcinoma
CDH11	Cadherin 11
COL3A1	Alpha 1 chain of collagen type III
COL6A1	Alpha 1 chain of collagen VI
COL6A2	Alpha 2 chain of collagen type VI
COL6A3	Alpha 3 chain of collagen type VI
CTGF	Connective tissue growth factor
E-cadherin	Epithelial cadherin
ECM	Extracellular matrix
EMT	Epithelial-to-mesenchymal transition
FN	Fibronectin
GAS1	Growth arrest specific 1
IL	Interleukin
INHBA	Inhibin beta A subunit
HTRA1	High-temperature requirement A1
MDSCs	Myeloid-derived suppressor cells
MMP2	Matrix metalloproteinase
NK	Natural killer (cells)
OSCC	Oral squamous cell carcinoma
PCOLCE	Procollagen C-proteinase enhancer protein
PD-L1	Programmed death-ligand 1
PDGFRA	Platelet-derived growth receptor alpha
RTV	Ritonavir
SPARC	Secreted protein acidic and rich in cysteine
TCGA	The cancer genome atlas
TGF	Transforming growth factor
TGM2	Transglutaminase 2
TREM2	Triggering receptor expressed on myeloid cells 2
VIM	Vimentin
