# Supplementary material for: Fibronectin Is a Likely Therapeutic Target Shared by Oral and Breast Carcinomas
Source: Int J Mol Sci. 2026 Jan 23;27(3):1148. doi: 10.3390/ijms27031148 (PMC12897945; doi:10.3390/ijms27031148)
Supplement: Supplementary file 1 [file ijms-27-01148-s001.zip › Supplementary Figures.pdf]

Supplementary Figures

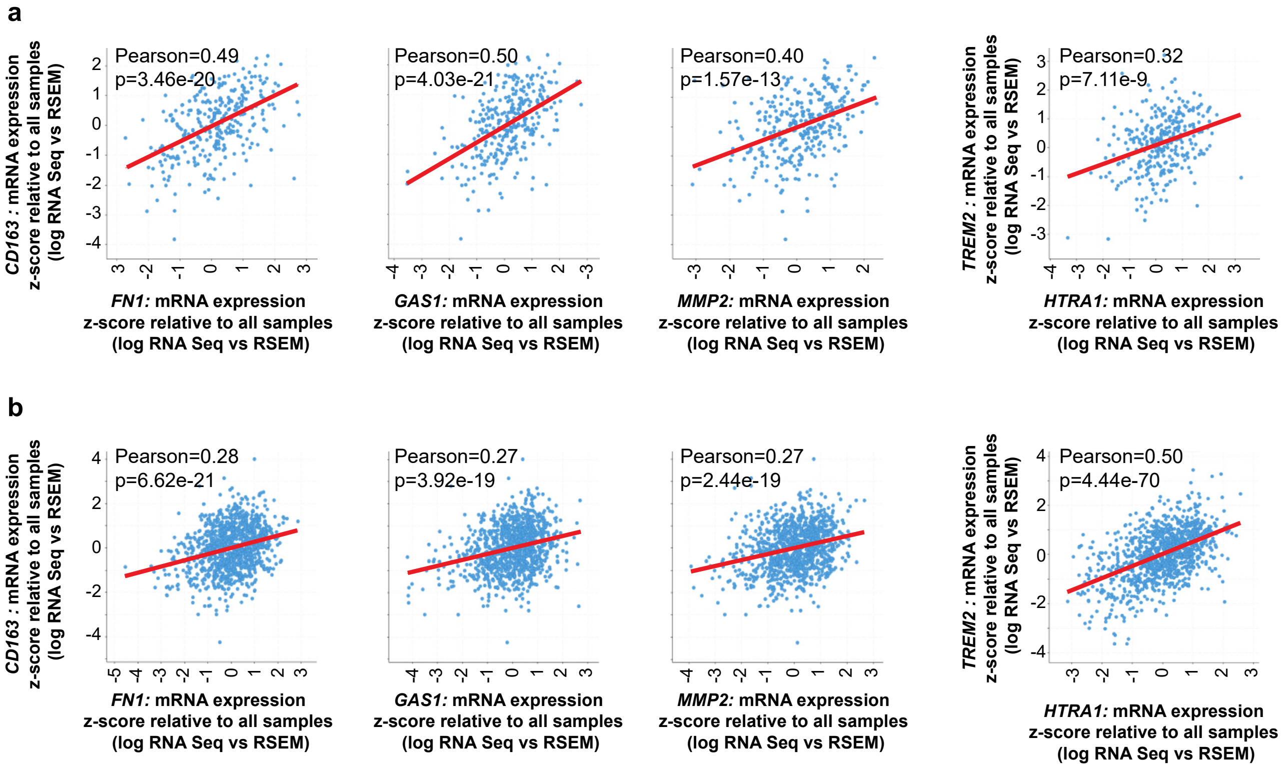

Supplementary Figure S1. Gene expression correlation of EMT genes with M2-like macrophages markers in TCGA BC dataset. Gene expression correlation have been computed in BC dataset between 13 EMT genes and **a)** *CD163* and **b)** *TREM2*. Red lines represent best-fit linear regressions. Exact r-values and p-values are reported in the figure.

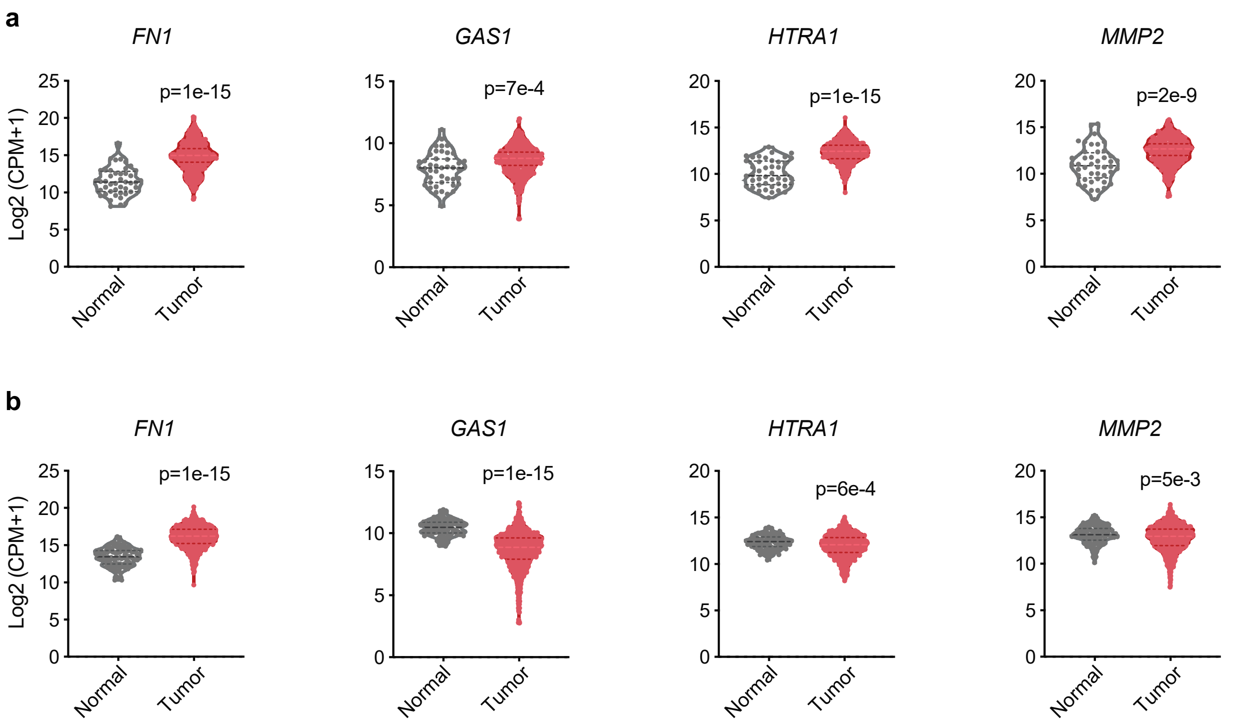

Supplementary Figure S2. Expression of EMT genes and proteins in normal and tumor tissues of OSCC and BC patients. a) Gene expression levels of *FN1*, *GAS1*, *HTRA1* and *MMP2* were compared between normal (n=44) and OSCC tissues (n=316) (TCGA cohort). **b)** Gene expression levels of *FN1*, *GAS1*, *HTRA1* and *MMP2* were compared between normal (n=114) and BC tissues (n=1097) (TCGA cohort). Exact p-values, calculated using two-tailed unpaired t-test, are reported in the figure. CPM: counts per million mapped reads.

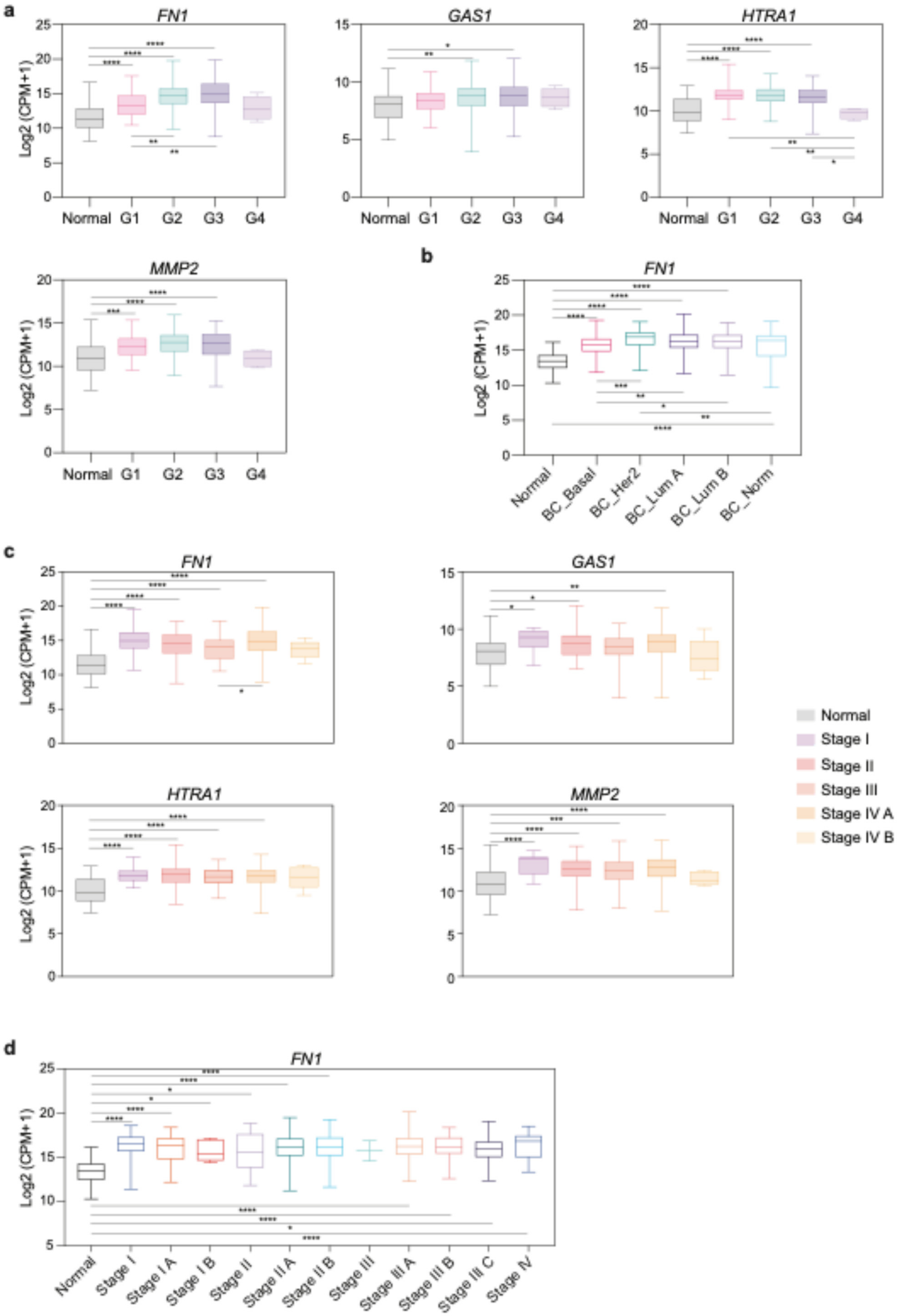
a
FN1
GAS1
HTRA1
MMP2
Log2 (CPM+1)
Normal
G1
G2
G3
G4
b
FN1
Log2 (CPM+1)
Normal
BC_Basal
BC_Her2
BC_Lum A
BC_Lum B
BC_Norm
c
FN1
GAS1
HTRA1
MMP2
Log2 (CPM+1)
Normal
Stage I
Stage II
Stage III
Stage IV A
Stage IV B
d
FN1
Log2 (CPM+1)
Normal
Stage I
Stage I A
Stage I B
Stage II
Stage II A
Stage II B
Stage III
Stage III A
Stage III B
Stage III C
Stage IV

Supplementary Figure S3. RNA-seq–based expression analysis of gene signature in OSCC and BC patients. a) Gene expression levels of *FN1*, *GAS1*, *HTRA1* and *MMP2* were compared among normal (n=44) and OSCC samples (TCGA cohort) stratified by tumor grade (G1 n=50; G2 n=189; G3 n=64; G4 n=4). **b)** Gene expression levels of *FN1* were compared among normal (n=114) and BC samples (TCGA cohort) stratified by molecular subtype (Basal-like n=171; HER2-enriched n=78; Luminal A n=499; Luminal B n=197; Normal-like n=36). **c)** Gene expression levels of *FN1*, *GAS1*, *HTRA1* and *MMP2* were compared among normal (n=44) and OSCC samples (TCGA cohort) stratified by tumor stage (I n=18; II n=54; III n=60; IV A n=149; IV B n=5). **d)** Gene expression levels of *FN1* were compared among normal (n=114) and BC samples (TCGA cohort) stratified by tumor stage (I n=89; I A n=86; I B n=5; II n=6; II A n=355; II B n=170; III n=2; III A n=155; III B n=28; III C n=64; IV n=19). One-way ANOVA for multiple comparisons, Turkey correction. * $p < 0.05$, ** $p < 0.01$, *** $p < 0.001$, **** $p < 0.0001$. Groups without statistical significance were unmarked. CPM; counts per million mapped reads.
